# Suicide and occupation: spatial patterns in Chapecó (SC) and contextualization of the temporal trend in the national scenario, 2006–2024

**DOI:** 10.1590/1980-549720260030

**Published:** 2026-07-27

**Authors:** Gabriela Rebeschini, Paulo Roberto Barbato, Jane Kelly Oliveira Friestino, Daniel Hideki Bando, Joanna d’Arc Lyra Batista

**Affiliations:** I Universidade Federal da Fronteira Sul – Chapecó (SC), Brazil.; II Universidade Federal de Santa Catarina, Speech-Language Pathology Department – Florianópolis (SC), Brazil.; III Universidade Federal de Alfenas, Instituto de Ciências da Natureza – Alfenas (MG), Brazil.; IV Universidade Federal de Ciências da Saúde de Porto Alegre, Department of Public Health – Porto Alegre (RS), Brasil.

**Keywords:** Suicide, Mortality, Spatio-temporal analysis, Employment, Public health

## Abstract

**Objective:**

To analyze suicide in Chapecó (SC) in terms of temporal trends, occupation, and spatial patterns by neighborhood, comparing local data with national data.

**Methods:**

This is an ecological study conducted in Chapecó (SC), based on deaths by suicide reported between 2006 and 2024. Suicide rates in Chapecó (SC) were standardized by age. The joinpoint regression technique was used for the temporal trend analysis. In the spatial analysis, the local empirical Bayesian suicide rate was calculated by neighborhood.

**Results:**

A total of 392 deaths were recorded during the period in Chapecó (SC). The average standardized annual rate was 8.8 per 100,000 inhabitants. There was a significant increase over the period studied in Brazil and in Chapecó (SC) (p<0.001), with more pronounced growth in this municipality. The profession of bricklayer/construction worker was the most reported one (13.6%), followed by agricultural workers (11.0%). The extreme south and north regions of the municipality had the highest suicide rates, above 9.6 deaths per 100,000 inhabitants.

**Conclusion:**

There was an upward trend in suicide in the municipality during the analyzed period. The results suggest a direct association between suicide and social deprivation and point to the need for intensified prevention efforts targeting construction workers and farmers, as well as the outlying areas of the city.

## INTRODUCTION

Suicide is considered a serious public health problem, characterized by its complex, multifactorial nature involving social, environmental, psychosocial, and historical conditions^1^. This phenomenon requires a careful, ongoing approach, since prevention measures can be implemented through the recognition of associated risk factors^1^. Globally, suicide shows high mortality rates, ranking as the fourth leading cause of death among people aged 15 to 29 years^2^. In Brazil, the South and Southeast regions stand out with the highest rates of self-harm/suicide attempts, making it essential to understand the characteristics and contexts surrounding suicide in order to develop effective prevention strategies^3^.

In a study of spatial patterns in Brazil from 1990-2015, high suicide rates were observed in the country’s South, which may be related to a complex combination of sociocultural, economic, and psychobiological factors – particularly among farmers from Rio Grande do Sul, the most affected population^4,5^.

In 2019, Santa Catarina had the second-highest suicide mortality rate in Brazil^3^, and in 2022 its western region recorded the highest suicide rate among the state’s regions^6^.

Thus, by recognizing incidence rates and their epidemiological and psychosocial characteristics, a strategy to reduce suicidal events that takes into account indicators of risk and protection can be developed^7^. Therefore, this study aims to analyze suicide in Chapecó (SC) with respect to temporal trends, occupation, and spatial patterns by neighborhood, comparing local data with national figures. Comparing Chapecó’s rates with national data is important to highlight the municipality’s particularities – it has a high life expectancy at birth and is one of the country’s main agroindustrial hubs, factors that may influence suicide rates.

## METHODS

### Design

**Figure 1:**
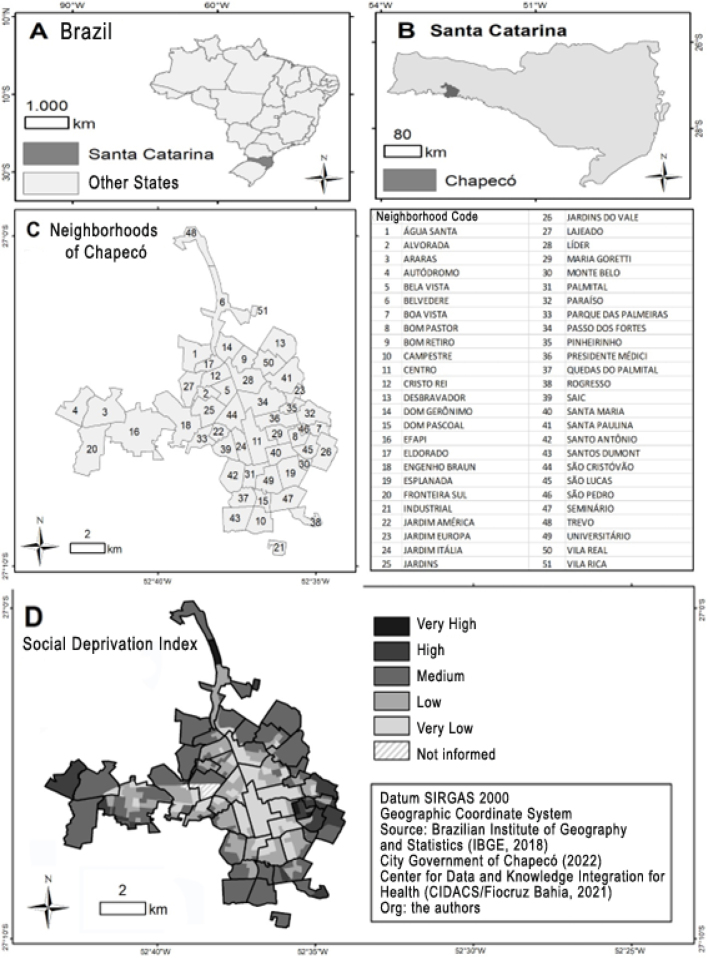
Map showing the location of Chapecó (SC) and the Social Deprivation Index.

Source: Prepared by the authors using data from the Brazilian Institute of Geography and Statistics (IBGE) and the Municipal Government of Chapecó.

This is an exploratory ecological spatiotemporal study of suicide, covering the period from January 2006 to December 2024 in the municipality of Chapecó, western Santa Catarina (Figure 1B).

### Context

This municipality had a population of 254,785 inhabitants and an area of 624.85 km2 in 2022^8^. The urban area is divided into 51 neighborhoods (Figure 1C)^9^. Figure 1D shows the mapping of the Brazilian Social Deprivation Index^10^. This is a synthetic sociodemographic indicator based on variables from the 2010 demographic census. It was calculated using information on income, education, and household sanitation conditions. The territorial unit is the census tract. The index was categorized into quintiles, with the first representing “very low” social deprivation and the fifth “very high.” Peripheral neighborhoods presented higher social deprivation, while the central region showed lower deprivation.

### Variable and data sources

The study population comprised all deaths of residents recorded in the Mortality Information System (SIM) for the municipality of Chapecó (SC) with suicide as the underlying cause, classified according to the International Classification of Diseases, 10th Revision (ICD-10) codes X60-X84, as available on the Department of Informatics of the Unified Health System (DataSUS) website^11^. In addition, data on occupational information and neighborhood of residence for suicide deaths from 2006 to 2019 were collected from the municipal epidemiological surveillance service of the Chapecó (SC) Municipal Health Secretariat in 2022. Suicide deaths in Brazil were obtained under the same conditions as those for Chapecó (SC), except that individual data on occupation and neighborhood of residence were not available. Population estimates were retrieved from DataSUS via TabNet. All data were organized in Google spreadsheets.

The variables studied were: type of event, sex, age (grouped into the age ranges 0–19, 20–39, 40–59, 60–79, and 80 years and over), marital status (single, married, and widowed), education (up to 3 years, 4–11 years, and 12 or more years of schooling), occupational group (classified according to Major Groups 01 to 09 of the Brazilian Classification of Occupations), and neighborhood of residence.

The suicide rate was calculated by dividing the number of suicide deaths in a given place and period by the corresponding population in that place and period, and then multiplying by 100,000 inhabitants per year. Next, a trend analysis of age-standardized suicide rates was performed using the 2010 Brazilian standard population and the direct method. This standardization was necessary for comparison purposes, as it removes the effect of age structure and allows for comparison of the “real risk” of death between Chapecó (SC) and Brazil.

### Statistical methods

The temporal trend analysis was performed using the Prais–Winsten procedure for generalized linear regression. To analyze the distribution of occupations among suicide deaths, absolute frequencies were obtained and relative frequencies were calculated in relation to the total number of suicide deaths in the period.

The temporal trend was analyzed using Poisson joinpoint regression (Joinpoint Regression Program, version 4.9.0.0). After defining the model, the annual percentage change was estimated to assess statistical significance, considering the null hypothesis of zero variation. Ninety-five percent confidence intervals (95% CI) were presented. The model used the Grid Search Method with zero to three joinpoints, and the Annual Percent Change (APC) and Average Annual Percent Change (AAPC) were estimated, with their CIs defined by the Parametric Method.

In the spatial analysis, the map showing the boundaries of neighborhoods in the urban area of Chapecó (SC) was generated based on a vector file available in the Chapecó (SC) Municipal Master Plan^12^. The file was georeferenced and converted into shapefile format. Neighborhood-level population data were based on the 2010 demographic census of the Brazilian Institute of Geography and Statistics (IBGE)^8^. The shapefile of Chapecó’s census tracts was downloaded and merged with the corresponding spreadsheet containing population data. Points were generated and randomly distributed according to the population of each census tract. The neighborhood layer was then added, and the points were counted within each neighborhood. National data were used only to contextualize the temporal trend; no spatial or occupation-based analyses were conducted for Brazil.

Given that suicide is a rare phenomenon, when it occurs in small populations it can lead to high instability in the calculation of mortality rates^13^. In addition, in Chapecó (SC), 25 neighborhoods had no recorded suicides during the study period. To minimize these instabilities, the local empirical Bayesian rate was calculated using the GeoDa 1.22 software. This technique computes a weighted average between the rate of each area and those of its neighboring areas, with weights proportional to the underlying population at risk. Areas with small populations tend to have substantially adjusted rates, whereas for larger populations the rates change very little^14^. All maps were produced using ArcGIS 10.6.

O processamento e análise dos dados foram realizados nos programas Microsoft Office Excel, versão 2016, e *Stata*, versão 12.0 (Stata-Corp LP, College Station, TX).

### Ethical aspects

The study complied with the ethical standards for research set forth in National Health Council Resolution No. 466 of 2012. The project was approved by the Research Ethics Committee under opinion No. 3,907,939/2020.

#### Data availability statement:

The full dataset supporting the findings of this study is available from the corresponding author upon reasonable request. The dataset is not publicly available due to the data integration procedures carried out by the authors.

## RESULTS

The municipality of Chapecó (SC) recorded a total of 392 suicide deaths between 2006 and 2024. The annual crude rates ranged from 4.8 (in 2010) to 14.9 (in 2022) suicides per 100,000 inhabitants. The average annual suicide rate in Chapecó was 9.1 per 100,000 inhabitants, and the average age-standardized rate was 8.8 deaths per 100,000 inhabitants.

Most suicides recorded in Chapecó (SC) were by hanging, strangulation, and suffocation (ICD-10, X70), accounting for 74.2% of suicide deaths between 2006 and 2024, a higher percentage than that observed in Brazil during the same period (67.2%).

**Figura 2:**
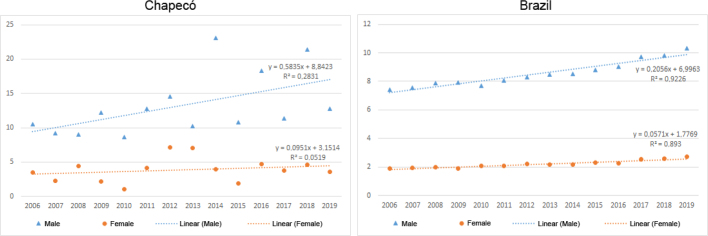
Suicide mortality trend, stratified by sex, in the municipality of Chapecó (SC) and in Brazil, 2006–2024.

Source: Prepared by the authors.

According to the data collected, the suicide trend stratified by sex in the municipality of Chapecó (SC) shows a greater increase among males than females. The same pattern is seen in Brazil, with higher male than female rates and a more pronounced upward trend among men (Figure 2).

When the trend by age group is analyzed in the municipality of Chapecó (SC), using the population divided by age strata, increasing trends are observed from age 20 onward, becoming more pronounced from age 60, especially among those aged 80 years or older. However, it is important to note that in the 80+ group there is high variability in the values due to the smaller population size. In Brazil, the linear trend in the 80+ group over the same period showed a slight increase, with the highest rate in the period recorded in 2021 (Supplementary Figure 1).

By educational level, the highest suicide rates in Chapecó (SC) were observed in the group with 4 to 11 years of schooling, as was also found for Brazil as a whole. The trend in this group was also one of greater increase (Supplementary Figure 2). Regarding trends by marital status, from 2006 to 2009 in Chapecó (SC), married individuals showed higher suicide rates. From 2010 onward, however, single individuals experienced increased suicide mortality, with a markedly rising linear trend. In Brazil, single individuals had higher trends than married and widowed individuals throughout the entire period analyzed (Supplementary Figure 3).

**Figura 3:**
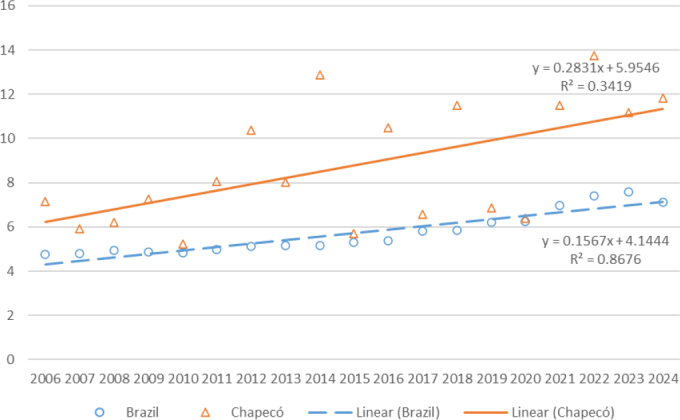
Linear regression of suicide mortality trend in Chapecó (SC) and in Brazil (per 100,000 inhabitants).

Source: Prepared by the authors.

The temporal trend analysis of suicide rates using the Prais–Winsten method showed a significant increase (p<0.001) both in Brazil and in Chapecó (SC), with a more pronounced rise in the latter. Despite greater variability in the rates due to its smaller population, Chapecó (SC) presents a clearly higher upward trend in suicide rates compared with Brazil (Figure 3).

Table 1 presents the temporal trend with the annual percent change in suicide rates in the total population and by sex in Brazil and in the municipality of Chapecó (SC). A significant increasing trend in suicide was observed in Brazil up to 2022, more evident between 2016 and 2022. Between 2022 and 2024, there is a non-significant decreasing trend in suicide rates in Brazil. In Chapecó (SC), there is a significant increasing trend in suicide rates throughout the entire period analyzed (2006–2024).

**Table 1:** Temporal trend analysis by joinpoint regression of age-adjusted suicide rates using the 2010 Brazilian standard population, in Brazil and in the municipality of Chapecó (SC), 2006–2024.

Type	Trend			Entire period		
	Period	APC	95%CI	AAPC	95%CI
Brazil	2006–2016	1.3^*^	0.7–1.8	2.4^*^	1.7–3.1
	2016–2022	5.5^*^	4.2–6.8		
	2022–2024	-0.7	-5.7–4.6		
Brazil – male gender	2006–2016	1.2^*^	0.6–1.7	2.3^*^	1.6–3.0
	2016–2022	5.2^*^	3.8–6.5		
	2022–2024	-0.7	-5.7–4.6		
Brazil – female gender	2006–2016	1.4^*^	0.5–2.2	2.7^*^	1.6–3.9
	2016–2022	6.5^*^	4.3–8.7		
	2022–2024	-1.2	-9.0–7.4		
Chapecó	2006–2024	3.3^*^	0.9–5.7	3.3 ^*^	0.9–5.7
Chapecó – male gender	2006–2024	3.1^*^	0.7–5.7	3.1^*^	0.7–5.7
Chapecó – female gender	2006–2024	3.1^*^	-0.8–7.2	3.1^*^	-0.8–7.2

APC: annual percent change; 95%CI: 95% confidence interval; AAPC: average annual percent change.

* Significantly different from zero (p<0.05).

Source: Prepared by the authors.

Between 2006 and 2019, of the 236 suicides in Chapecó (SC), 199 (84.3%) had recorded occupational information. Table 2 presents the distribution of deaths according to the major groups of the Brazilian Classification of Occupations, with the most prevalent occupations listed as subitems within each group.

**Table 2:** Distribution of occupations among suicide deaths of Chapecó residents (SC), 2006-2019 (n=199).

Major Groups of the Brazilian Classification of Occupations	n (%)	n
Major Group 0^*^	1 (0.5)	
Major Group 1^†^	4 (2.1)	
Retailer		3
Major Group 2^‡^	7 (3.5)	
Major Group 3^§^	13 (6.6)	
Major Group 4^//^	26 (13.1)	
Production tracker		20
Major Group 5^¶^	21 (10.2)	
Domestic worker – general services		6
Major Group 6^#^	22 (11.1)	
Farm caretaker		14
Seasonal farmworker		8
Major Groups 7 and 8^**^	58 (29.2)	
Bricklayer		23
Construction helper		4
Mechanical fitter		7
Truck driver		5
Major Group 9^††^	1 (0.5)	
No occupation reported	46 (23.2)	
Student		11
Housekeeper		10
Retiree / pensioner		17
Chronically unemployed		8

*Members of the armed forces, police officers, and military firefighters;

^†^Senior public officials; leaders of public-interest organizations and companies; managers;

^‡^Science and arts professionals;

^§^Mid-level technicians;

^//^Administrative staff;

^¶^ Service workers; shop and market salespersons;

^#^ Agricultural, forestry, and fishing workers;

^**^Industrial goods and services production workers;

^††^Repair and maintenance service workers.

Source: Prepared by the authors.

The most prevalent occupational group (29.2%) was industrial goods and services production workers, with bricklayers being the most frequently recorded occupation (23 notifications, rising to 27 if construction helpers are included). The second most prevalent occupation among suicides was agricultural workers, with 22 reported cases (14 caretakers and 8 seasonal farmworkers). Third, we identified production timekeepers, with 20 notifications. The group without a defined occupation (students, housewives, retirees/pensioners, and chronically unemployed individuals) accounted for 23.2% of suicide cases with recorded occupation.

**Figure 4:**
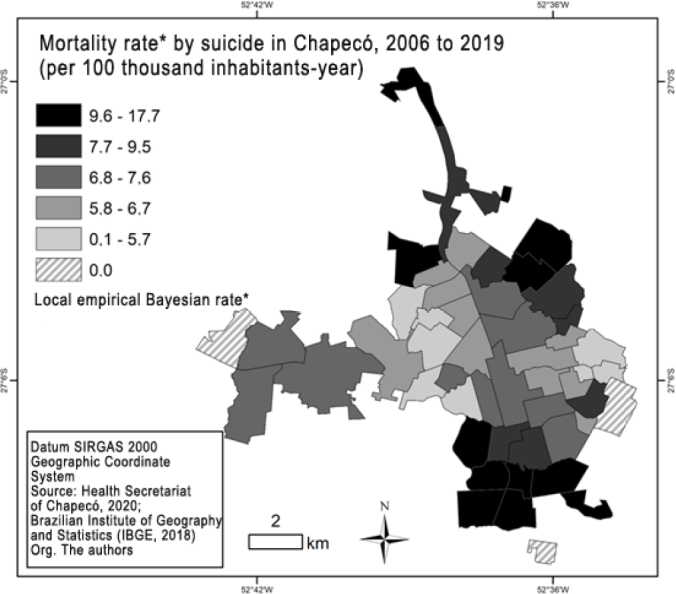
Suicide rate in Chapecó (SC), 2006–2019.

Source: Prepared by the authors, using data from the Brazilian Institute of Geography and Statistics (IBGE) and the Chapecó Municipal Health Secretariat.

The map in Figure 4 shows the spatial distribution of suicide rates in Chapecó (SC). In the far southern part of the municipality, there was a cluster of seven neighborhoods with high rates, above 9.6 deaths per 100,000 inhabitants. In the northern area, some neighborhoods also showed rates within this range. The result suggests an association between suicide and high social deprivation.

## DISCUSSION

The average annual age-standardized suicide rate in Chapecó (SC) showed an increasing trend over the study period, which was higher than the national trend (p<0.001). Most suicides were due to hanging, and the characteristics with the greatest increasing trend were male sex, age 80 years or older, 4 to 11 years of schooling, and being single. In Chapecó (SC), the occupation of bricklayer/construction helper was the most frequently reported between 2006 and 2019, followed by agricultural workers, with cases more concentrated in areas of high social deprivation.

Historically, the southern states have shown higher suicide rates than the national average, with a greater concentration of deaths in this region^3,15,16^. In Santa Catarina, the microregion of Chapecó (SC) records a suicide burden higher than the state average^17^.

Most suicide deaths reported between 2006 and 2024 in Chapecó (SC) were due to hanging, strangulation, and suffocation. Choi et al. report that the method used to commit suicide may be associated with sex and age^18^. It has been observed that older men choose more violent and lethal methods, such as hanging and firearm use, whereas women and young people tend to choose subtler and slower methods, such as jumping from heights and poisoning.

In a study conducted in Rio Grande do Sul, hanging was the most commonly used method for suicide^19^. It is noteworthy that access to this method is difficult to control, making it a risk predictor for self-directed violence. This underscores the importance of early identification of vulnerable populations for preventive measures, thereby helping to avoid progression to the fatal act.

Minayo et al.^20^, in a study analyzing the epidemiological profile of older men who died by suicide in Brazil, divided them into three occupational branches: the highest percentage was in agriculture, followed by services such as bricklaying and carpentry, which corroborates the findings of the present study. They also report that men raised and socialized under hegemonic masculinity norms see themselves as guardians and may view suicide as an honorable way out of financial debt, a situation that is highly prevalent among those working in agriculture.

Regarding sex, the results showed a higher suicide trend in the male population, accounting for about 77% of suicides in the city of Chapecó (SC). This finding is consistent with a study conducted in the city of Marabá (PA)^21^. The findings are in line with what is observed at the national level and may be explained by the greater lethality of the methods chosen to complete the act.

Another important factor in the persistently higher suicide rates among men is socioeconomic conditions, including instabilities and impulsive, competitive behaviors associated with alcohol use, which may become predictors of suicidal acts^22^.

Regarding age group, we identified in Chapecó (SC) an upward trend starting at 20 years of age, with a steeper increase from 60 years onward and a marked presence among those aged 80 years or older. In the state of Rio Grande do Sul, during the period from 2010 to 2016, being male and over 45 years of age was found to be a predictor of suicide by hanging. Furthermore, in the period from 2009 to 2012, male sex remained predisposed to this method of suicide, in association with being single^16^. The main interconnected risk factors among these older adults may stem from physical illnesses, social isolation, family problems, inactivity, and the abusive use of psychoactive substances — especially in association with mental disorders such as depression. Depression appears to be the factor that most affects these adults and older adults, contributing to premature deaths^16^.

Most individuals had low to medium educational attainment (4–11 years of schooling), a pattern also observed at the national level^22^. Educational level, together with other social factors, is a key determinant of socioeconomic status, directly influencing daily worries and stress, and consequently affecting self-esteem and suicidal ideation^22^.

Regarding suicide trends by marital status, in the municipality of Chapecó (SC) married individuals had higher suicide rates up to 2009; however, from 2010 onward, single individuals showed increased rates, with a linear upward trend. In Brazil as a whole, single individuals consistently had higher trends than married and widowed individuals throughout the entire period analyzed. This finding in Chapecó (SC) differs from a study conducted in the state of Santa Catarina between 2014 and 2018^23^, in which married individuals accounted for 49.4% of suicide deaths, followed by single individuals with 34.7%; among the others, separated and divorced individuals accounted for 10.1% and widowed individuals for 5.5%.

Chapecó (SC) shows an upward trend in suicides that is higher than the national trend, possibly related to cultural and socioeconomic factors and to the predominance of agroindustry in the local economy. Between 2016 and 2022, suicide rates in Brazil also increased, a period marked by political instability and reforms that affected workers’ socioeconomic security. The loosening of labor relations, changes to the pension system, and austerity policies have contributed to a context of precarious work and reduced social protection. This phenomenon is consistent with international findings that link economic crises and austerity to increased suicide, particularly in the absence of social protection mechanisms, especially in low- and middle-income countries^24,25,26^.

In Brazil, this phenomenon may be related not only to unemployment, but also to working conditions and the loss of rights, factors that can increase psychological stress even among formally employed workers^27^. In turn, the decrease observed from 2022 onward still occurs in a late-pandemic context, as evidenced in other studies^28,29^.

A study conducted in the Legal Amazon region, characterized by livestock farming, monocultures, and extractive activities, identified higher suicide rates in rural areas^30^. The intensive use of pesticides in this production model reinforces the association between agricultural activities, occupational exposure, and a higher occurrence of suicide^30,31^. Although further studies are needed to assess causal relationships, such ecological data in regions with agricultural production hubs, as in the case of the municipality of Chapecó (SC), may suggest a possible association between work and exposure to agricultural disruptors.

Pesticides can lead to suicide either directly or indirectly^31^. Directly, when they are ingested or intentionally inhaled with the purpose of dying; and indirectly, through improper use that affects the nervous system and contributes to the development of mental disorders, triggering neuropathologies and, in turn, suicidal ideation^32^.

When analyzing the occupational profiles of suicide deaths over the study period, a higher percentage was observed among individuals working in construction, especially bricklayers (29.2%); this was followed by individuals without a defined occupation (23.2%), such as students, housewives, unemployed persons, retirees, or pensioners; individuals employed as production timekeepers (13.1%); and agricultural workers, who accounted for 11.1% of cases.

Regarding individuals without a defined occupation, it is important to note that retirees generally have prior work experience or continue working to supplement their income. As for the “production timekeeper” classification, it could not be further detailed because it does not objectively specify the role, although it is assumed to be related to workers in agroindustry, especially large slaughterhouses located in the municipality.

The findings are consistent with the literature in indicating a higher occurrence of suicide deaths among agricultural and construction workers^33,34^. These occupations are recurrently identified as being at greater risk for suicidal behavior, especially in contexts of precarious work, economic instability, and high physical and psychosocial demands.

Suicide among workers in sectors marked by precarious labor conditions, such as agriculture and construction, has been described in studies on the socio-occupational profile of deaths. In these occupations, vulnerable working conditions can intensify mental distress and increase the risk of suicide, a phenomenon observed in both national and international contexts^35,36,37^.

According to Tornesi^38^, moral harassment in the construction sector is widespread and, as a factor with health consequences, requires consideration of psychological harm, which may include distress, anxiety, and persistent sad thoughts. Harassment can lead to traumatic situations in which the targeted worker is unable to overcome what happened, potentially resulting in drug use, especially alcohol. The suffering that is imposed and not understood may lead to suicidal ideation; however, this is not limited to an individual phenomenon, but rather a social process stemming from capitalist modes of production^39^.

This study has limitations inherent to its ecological design, such as the inability to infer individual causality. The lack of detailed occupational information restricted analyses to comparing trends in occurrence; in addition, possible inconsistencies and underreporting may have reduced internal validity. To minimize these effects, we adopted variable standardization strategies and checked the consistency of records. Despite these limitations, the findings contribute to identifying possible factors associated with suicide and to guiding future investigations and preventive strategies targeting at-risk populations.

In this sense, the trends observed in the municipality help to outline a profile of individuals with a higher likelihood of dying by suicide: men, older adults, construction and/or agricultural sector workers, single individuals, and residents of neighborhoods with higher social deprivation.

It is important to emphasize that, with regard to construction work — particularly bricklayers — there are few studies specifically focused on this occupational group, even though this profession also appears with high frequency in the results of other studies.
