# ERRATUM: Suicide and occupation: spatial patterns in Chapecó (SC) and contextualization of the temporal trend in the national scenario, 2006–2024

**DOI:** 10.1590/1980-549720260030erratum

**Published:** 2026-10-05

In the manuscript "*Suicide and occupation: spatial patterns in Chapecó (SC) and contextualization of the temporal trend in the national scenario, 2006–2024*", DOI: https://doi.org/10.1590/1980-549720260030, published in Rev Bras Epidemiol. 2026; 29: e260030.

**Where it reads:**

**FUNDING:** none.

**It should read:**

**FUNDING:** Universidade Federal da Fronteira Sul - PES-2025-0260.

