# Supplementary material for: Suicide and occupation: spatial patterns in Chapecó (SC) and contextualization of the temporal trend in the national scenario, 2006–2024
Source: Rev Bras Epidemiol. 2026 Jul 27;29:e260030. doi: 10.1590/1980-549720260030 (PMC13404845; doi:10.1590/1980-549720260030)

Figura Suplementar 1. Tendência de mortalidade por suícidio, estratificada por faixa etária, no município Chapecó e no Brasil, 2006-2025.

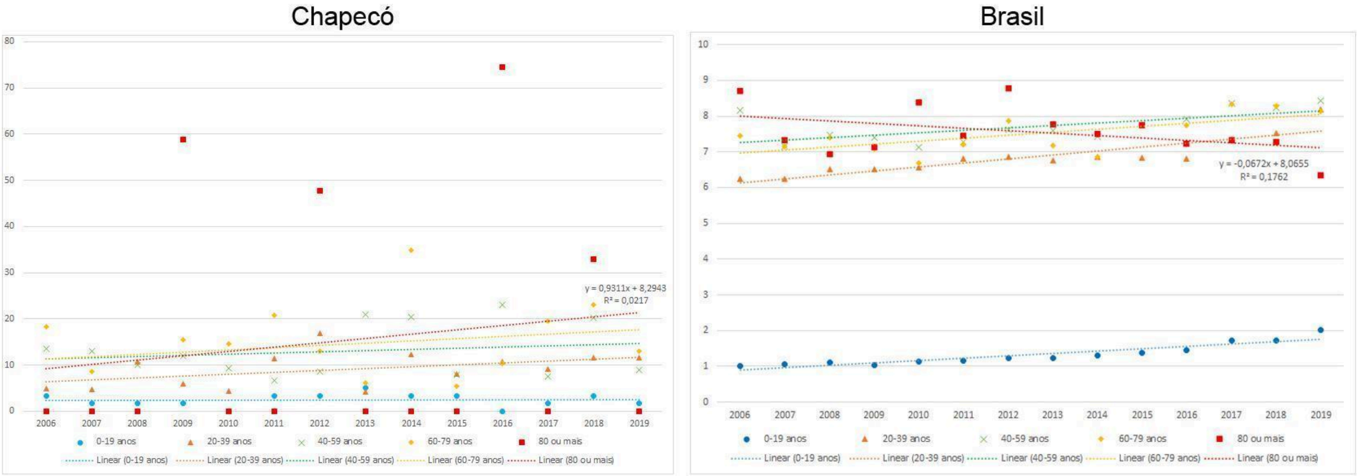

Figura Suplementar 2. Tendência de mortalidade por suícidio, estratificada por anos de estudo, no município Chapecó e no Brasil, 2006-2019.

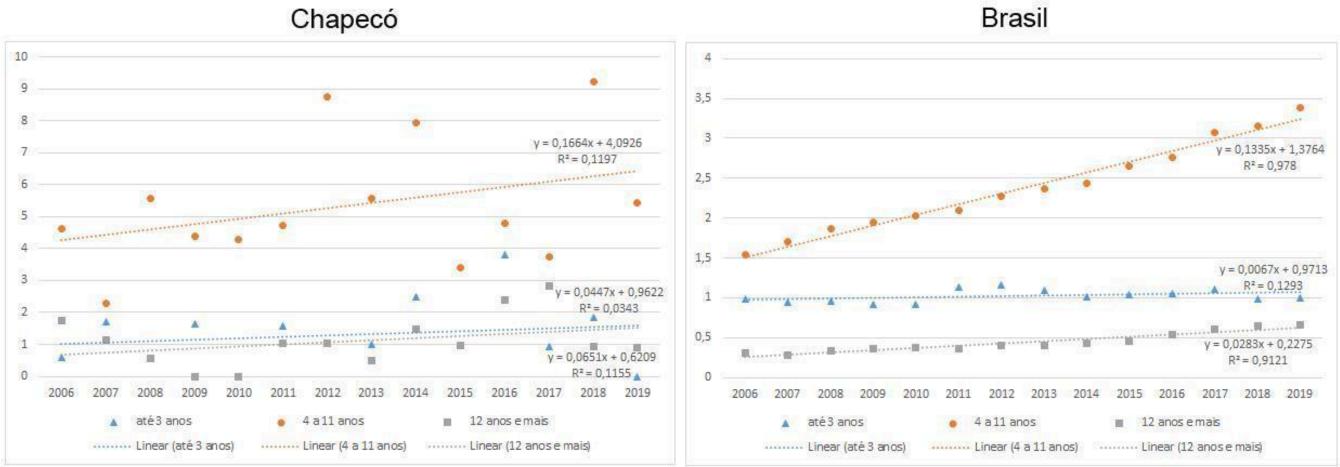

Figura Suplementar 3. Tendência de mortalidade por suícidio, estratificado por estado civil, no município Chapecó e no Brasil, 2006-2019.

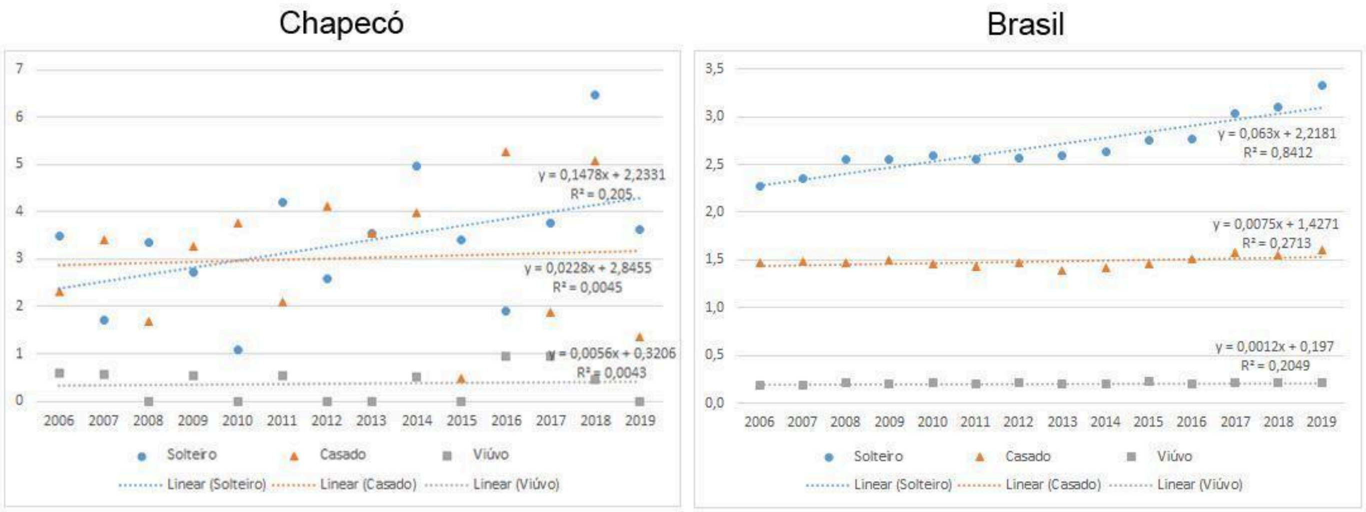

Supplement: Supplementary Figure 1 [file 1980-5497-rbepid-29-e260030-supp1.pdf]
