# Supplementary material for: Suicide and occupation: spatial patterns in Chapecó (SC) and contextualization of the temporal trend in the national scenario, 2006–2024
Source: Rev Bras Epidemiol. 2026 Jul 27;29:e260030. doi: 10.1590/1980-549720260030 (PMC13404845; doi:10.1590/1980-549720260030)
Supplement: Translation

# Translation: Suicídio e ocupação: padrões espaciais em Chapecó (SC) e contextualização da tendência temporal no cenário nacional, 2006–2024

**DOI:** 10.1590/1980-549720260030.2

**Authors:** Gabriela Rebeschini, Paulo Roberto Barbato, Jane Kelly Oliveira Friestino, Daniel Hideki Bando, Joanna d’Arc Lyra Batista

**Affiliations:** I Universidade Federal da Fronteira Sul – Chapecó (SC), Brasil.; II Universidade Federal de Santa Catarina, Departamento de Fonoaudiologia – Florianópolis (SC), Brasil.; III Universidade Federal de Alfenas, Instituto de Ciências da Natureza – Alfenas (MG), Brasil.; IV Universidade Federal de Ciências da Saúde de Porto Alegre, Departamento de Saúde Coletiva – Porto Alegre (RS), Brasil.

**Keywords:** Suicídio, Mortalidade, Análise espaço-temporal, Emprego, Saúde pública

## Abstract

**Objetivo:**

Analisar o suicídio em Chapecó (SC) quanto à tendência temporal, à ocupação e ao padrão espacial por bairros, comparando os dados locais com os nacionais.

**Métodos:**

Trata-se de estudo ecológico realizado em Chapecó (SC), com base em óbitos por suicídio notificados entre 2006 e 2024. As taxas de suicídio de Chapecó (SC) foram padronizadas por idade. Para a análise de tendência temporal, usou-se a técnica de regressão joinpoint. Na análise espacial foi calculada a taxa de suicídio bayesiana empírica local por bairros.

**Resultados:**

Foram registrados 392 óbitos no período em Chapecó (SC). A taxa anual média padronizada foi de 8,8 por 100 mil hab. Evidenciou-se aumento significativo ao longo do período estudado no Brasil e em Chapecó (SC) (p<0,001), com crescimento mais acentuado neste município. A profissão de pedreiro/servente de obras foi a mais notificada (13,6%); em segundo lugar, os trabalhadores da agricultura (11%). As regiões extremo sul e norte do município de Chapecó (SC) apresentaram as maiores taxas de suicídio, acima de 9,6 óbitos por 100 mil habitantes.

**Conclusão:**

Houve uma tendência de aumento de suicídio no município durante o período analisado. O resultado sugere associação direta do suicídio com a privação social e aponta para a necessidade e intensificação das ações de prevenção direcionadas aos trabalhadores da construção civil e agricultores, bem como para as regiões periféricas da cidade.

## INTRODUÇÃO

O suicídio é considerado um grave problema de saúde pública, caracterizado por sua natureza complexa e multi-fatorial, envolvendo condições sociais, ambientais, psicossociais e históricas^1^. Esse fenômeno exige uma abordagem cuidadosa e contínua, uma vez que medidas de prevenção podem ser implementadas por meio do reconhecimento dos fatores de risco associados^1^. Globalmente, o suicídio apresenta altas taxas de mortalidade, sendo a quarta maior causa de morte entre jovens de 15 a 29 anos^2^. No Brasil, destacam-se as regiões Sul e Sudeste, com os maiores índices de tentativas de autolesão, tornando-se fundamental compreender as características e os contextos que envolvem o suicídio, para que possam ser desenvolvidas estratégias eficazes de prevenção^3^.

Em estudo de padrões espaciais do Brasil dos anos de 1990-2015, verificou-se, na região Sul do país, altas taxas de suicídio, podendo estar relacionadas com complexa combinação de condicionantes socioculturais, econômicos e psicobiológicos — principalmente nos agricultores gaúchos, população mais afetada^4,5^.

Em 2019, Santa Catarina teve a segunda maior taxa de óbito por suicídio no Brasil (3), sendo a região oeste a que apresentou a maior taxa de suicídio dentre as regiões do estado em 2022^6^.

Deste modo, se reconhecendo as taxas de incidência e suas características epidemiológicas e psicossociais, pode-se construir uma estratégia de redução de eventos suicidas, que considere os indicativos de risco ou proteção^7^. Com isso, este estudo tem como objetivo analisar o suicídio em Chapecó (SC) quanto à tendência temporal, à ocupação e ao padrão espacial por bairros, comparando os dados locais com os nacionais. É importante comparar as taxas de Chapecó (SC) com os dados nacionais para evidenciar as particularidades do município, que é uma região com alta esperança de vida ao nascer e um dos principais polos agroindustriais do país, o que pode influenciar nas taxas de suicídio.

## MÉTODOS

### Delineamento

Trata-se de um estudo ecológico exploratório espaço-temporal de suicídio, sendo o período analisado entre janeiro de 2006 e dezembro de 2024 no município de Chapecó, região oeste de Santa Catarina (Figura 1B).

**Figura 1:** Mapa de localização de Chapecó (SC) e Índice de Privação Social.

Fonte: Elaboração própria, com uso de dados do Instituto Brasileiro de Geografia e Estatística e Prefeitura Municipal de Chapecó.

### Contexto

Este município apresentava uma população de 254.785 habitantes e área de 624,85 km^2^, no ano de 2022^8^. A área urbana é dividida em 51 bairros (Figura 1C)^9^. A Figura 1D representa o mapeamento do Índice Brasileiro de Privação Social^10^. Trata-se de um indicador sintético sociodemográfico baseado nas variáveis do censo demográfico de 2010. Ele foi calculado utilizando informações de renda, escolaridade e condições de saneamento do domicílio. A unidade territorial é o setor censitário. O índice foi categorizado em quintis, sendo o primeiro com o nível “muito baixo” de privação social e o quinto, como “muito alto”. Nota-se que os bairros das periferias apresentaram maior privação social e a região do centro, a menor.

### Variáveis e fontes de dados

A população de estudo foi composta pelo conjunto de óbitos por residência, notificados no Sistema de Informação de Mortalidade (SIM) para o município de Chapecó (SC), tendo como causa básica o suicídio — conforme a Classificação Internacional de Doenças-10 (CID-10), mediante os códigos X60 a X84, disponíveis no site Departamento de Informação e Informática do Sistema Único de Saúde (DataSUS)^11^. Além disso, foram realizadas coletas de informações sobre ocupação profissional e bairro de residência dos óbitos registrados por suicídio de 2006 a 2019, disponibilizados pelo serviço municipal de vigilância epidemiológica, da Secretaria Municipal de Saúde de Chapecó (SC), em 2022. Os óbitos por suicídio no Brasil foram levantados sob as mesmas condições dos que ocorreram em Chapecó (SC), exceto informações individuais sobre ocupação profissional e bairro de residência. As estimativas populacionais foram obtidas no DataSUS por meio do TabNet. Todos os dados foram organizados em planilhas eletrônicas do Google.

As variáveis estudadas foram: tipologia, sexo, idade (agrupadas nas faixas etárias de 0–19, 20–39, 40–59, 60–79, e 80 e mais), estado civil (solteiros, casados e viúvos), escolaridade (até 03 anos, 04 a 11 anos, e 12 anos e mais de estudo), ocupação profissional (classificados de acordo com os grandes Grupos de 01 a 09 da Classificação Brasileira de Ocupações) e bairro de residência.

A taxa de suicídio foi calculada dividindo-se o número de óbitos por suicídio, no local e período, pela população, no local e período multiplicado por 100 mil habitantes/ano. Em seguida, foi realizada a análise de tendência das taxas de suicídio padronizada por idade, com a população padrão do Brasil de 2010, pelo método direto. Essa padronização foi necessária para proceder com a comparação, pois elimina o efeito da estrutura etária, permitindo comparar o “risco real” de morte entre Chapecó (SC) e Brasil.

### Métodos estatísticos

A análise de tendência temporal foi realizada utilizando-se o procedimento de Prais-Winsten para regressão linear generalizada. Para análise da distribuição das ocupações dos óbitos por suicídio, foram levantadas a frequência bruta e calculada a frequência relativa em relação ao conjunto de óbitos por suicídio no período.

A tendência temporal foi analisada por regressão *joinpoint* (por pontos de inflexão) de Poisson (*Joinpoint Regression Program*, versão 4.9.0.0). Após a definição do modelo, estimou-se a variação percentual anual para avaliar significância estatística, considerando a hipótese nula de variação igual a zero. Foram apresentados os Intervalos de Confiança (IC) de 95%. O modelo utilizou o *Grid Search Method*, com zero a três joinpoints, sendo estimada a Mudança Anual Percentual (APC, na sigla em inglês) e a Mudança Anual Percentual Média (AAPC, na sigla em inglês), e seus IC definidos pelo Método Paramétrico.

Na análise espacial, o mapa com a delimitação dos bairros na área urbana de Chapecó (SC) foi gerado com base em um arquivo vetorial disponível no Plano Diretor Municipal de Chapecó (SC)^12^. O arquivo foi georreferenciado e convertido no formato *shapefile*. A população por bairro foi baseada nos dados do censo demográfico de 2010 do Instituto Brasileiro de Geografia e Estatística (IBGE)^8^. Foram baixados o *shapefile* dos setores censitários de Chapecó (SC) e unidos com a respectiva planilha com os dados populacionais. Foram gerados pontos distribuídos aleatoriamente de acordo com a população de cada setor. Posteriormente, foi adicionada a camada dos bairros, e os pontos foram contados em cada bairro. Os dados nacionais foram utilizados apenas para contextualização da tendência temporal, não sendo realizadas análises espaciais ou por ocupação no Brasil.

Dado que o suicídio é um fenômeno raro, quando ocorre em populações pequenas, pode acarretar altas instabilidades no cálculo das taxas de mortalidade^13^. Além disso, em Chapecó (SC), 25 bairros não apresentaram registro de suicídio no período estudado. Para minimizar essas instabilidades, foi calculada a taxa *bayesiana* empírica local com o programa Geoda 1.22. Essa técnica calcula a média ponderada entre a taxa de cada área e respectivas áreas vizinhas, com pesos proporcionais à população subjacente em risco. Áreas com populações pequenas tenderão a ter taxas consideravelmente ajustadas, enquanto para populações maiores as taxas mudarão pouco^14^. Todos os mapas foram elaborados no programa ArcGIS 10.6.

O processamento e análise dos dados foram realizados nos programas Microsoft Office Excel, versão 2016, e *Stata*, versão 12.0 (Stata-Corp LP, College Station, TX).

### Aspectos éticos

A pesquisa observou as normas sobre ética em pesquisa contidas na Resolução número 466 de 2012, do Conselho Nacional de Saúde. O projeto foi aprovado pelo Comitê de Ética em Pesquisa sob parecer número 3.907.939/2020.

#### Declaração de disponibilidade de dados:

Todo o conjunto de dados que dá suporte aos resultados deste estudo está disponível mediante solicitação ao autor correspondente. O conjunto de dados não está publicamente disponível devido à integração de bancos de dados realizados pelos autores.

## RESULTADOS

O município de Chapecó (SC), apresentou um total de 392 óbitos por suicídio entre os anos de 2006 e 2024. As taxas brutas anuais variaram de 4,8 (em 2010) a 14,9 (em 2022) suicídios por 100 mil habitantes. A taxa anual média de suicídio em Chapecó foi de 9,1/100 mil hab.; a taxa média padronizada, de 8,8 óbitos/100 mil hab.

A maioria dos suicídios registrados em Chapecó (SC) foi por enforcamento, estrangulamento e sufocação (CID-10, X70), que correspondeu a 74,2% dos óbitos por suicídio entre 2006 e 2024, percentual maior do que o encontrado no mesmo período no Brasil (67,2%).

Conforme os dados levantados, a tendência de suicídio estratificada por sexo no município de Chapecó (SC) demonstra uma tendência de aumento maior no sexo masculino em relação ao feminino. O mesmo comportamento se encontra no Brasil, com taxas masculinas maiores que as femininas e tendência de aumento mais acentuada (Figura 2).

**Figura 2:** Tendência de mortalidade por suicídio, estratificada por sexo, no município Chapecó (SC) e no Brasil, 2006–2024.

Fonte: Elaboração própria.

Quando se analisa a tendência por faixa etária no município de Chapecó (SC), população base dividida por faixa etária, observam-se tendências de aumento a partir dos 20 anos de idade, acentuando-se a partir de 60 anos, especialmente para 80 ou mais — porém, vale ressaltar que, no caso de 80 anos ou mais, há muita dispersão de valores devido ao menor número populacional. No caso do Brasil, a tendência linear no grupo de 80 anos ou mais no mesmo período foi de leve acréscimo, com a maior taxa do período registrada no ano de 2021 (Figura Suplementar 1).

De acordo com a escolaridade, os maiores coeficientes de suicídio em Chapecó (SC) estão no grupo de 4 a 11 anos de estudo, igualmente como observado no Brasil. A tendência nesse grupo também é de maior aumento (Figura Suplementar 2). Quanto às tendências de acordo com o estado civil, observou-se que, de 2006 a 2009, no município de Chapecó (SC), os casados apresentavam maiores coeficientes de suicídio. A partir de 2010, porém, os solteiros aumentaram as taxas de óbito por suicídio, com tendência linear de aumento bastante acentuada. No caso do Brasil, os solteiros tiveram tendência maior do que os casados e os viúvos durante todo o período analisado (Figura Suplementar 3).

A análise da tendência temporal das taxas de suicídio pelo método de Prais-Winsten indicou aumento significativo (p<0,001) tanto no Brasil quanto em Chapecó (SC), sendo a elevação mais acentuada nesta última; apesar da maior dispersão das taxas devido à população menor, Chapecó (SC) apresenta uma tendência de aumento nas taxas de suicídio nitidamente superior à do Brasil (Figura 3).

**Figura 3:** Regressão linear da tendência de óbito por suicídio em Chapecó (SC) e no Brasil (por 100 mil habitantes).

Fonte: Elaboração própria.

A Tabela 1 apresenta a tendência temporal com a variação percentual anual para as taxas de suicídio na população total e por sexo biológico do Brasil e do município de Chapecó (SC). Verificou-se uma tendência significativa de incremento no suicídio no Brasil até 2022, sendo mais evidente entre os anos de 2016 e 2022. Já entre 2022 e 2024 há uma tendência não significativa de decréscimo das taxas de suicídio no Brasil. No município de Chapecó (SC) há uma tendência significativa de aumento nas taxas de suicídio durante todo o período analisado (2006 a 2024).

**Tabela 1:** Análise de tendências temporais por regressão por pontos de inflexão (*joinpoints*) das taxas de suicídio, ajustadas por faixa etária com a população padrão do Brasil de 2010, no Brasil e no município de Chapecó (SC), 2006–2024.

Tipo	Tendência			Período inteiro	
	Período	APC	IC95%	AAPC	IC95%
Brasil	2006–2016	1,3^*^	0,7–1,8	2,4^*^	1,7–3,1
	2016–2022	5,5^*^	4,2–6,8		
	2022–2024	-0,7	-5,7–4,6		
Brasil sexo masculino	2006–2016	1,2^*^	0,6–1,7	2,3^*^	1,6–3,0
	2016–2022	5,2^*^	3,8–6,5		
	2022–2024	-0,7	-5,7–4,6		
Brasil sexo feminino	2006–2016	1,4^*^	0,5–2,2	2,7^*^	1,6–3,9
	2016–2022	6,5^*^	4,3–8,7		
	2022–2024	-1,2	-9,0–7,4		
Chapecó	2006–2024	3,3^*^	0,9–5,7	3,3 *	0,9–5,7
Chapecó sexo masculino	2006–2024	3,1^*^	0,7–5,7	3,1^*^	0,7–5,7
Chapecó sexo feminino	2006–2024	3,1^*^	-0,8–7,2	3,1^*^	-0,8–7,2

APC: variação percentual anual; IC95%: Intervalo de confiança a 95%; AAPC: variação percentual anual média.

*Significativamente diferente de zero (p<0,05).

Fonte: Elaboração própria.

Entre 2006 e 2019, dos 236 suicídios em Chapecó (SC), 199 (84,3%) tinham registro de ocupação. A Tabela 2 apresenta a distribuição dos óbitos segundo os grandes grupos da Classificação Brasileira de Ocupações, com as profissões mais prevalentes listadas como subitens de cada grupo.

**Tabela 2:** Distribuição das ocupações dos óbitos por suicídio de residentes em Chapecó (SC), 2006–2019 (n=199).

Grande Grupo da Classificação Brasileira de Ocupações	n (%)	n
Grande Grupo 0^*^	1 (0,5)	
Grande Grupo 1^†^	4 (2,1)	
Comerciante varejista		3
Grande Grupo 2^‡^	7 (3,5)	
Grande Grupo 3^§^	13 (6,6)	
Grande Grupo 4^//^	26 (13,1)	
Apontador de produção		20
Grande Grupo 5^¶^	21 (10,2)	
Empregado doméstico nos serviços gerais		6
Grande Grupo 6^#^	22 (11,1)	
Caseiro (agricultura)		14
Trabalhador volante da agricultura		8
Grandes Grupos 7 e 8^**^	58 (29,2)	
Pedreiro		23
Servente de obras		4
Ajustador mecânico		7
Motorista de caminhão		5
Grande Grupo 9^††^	1 (0,5)	
Sem ocupação definida	46 (23,2)	
Estudante		11
Dona de casa		10
Aposentado/pensionista		17
Desempregado crônico		8

*Membros das forças armadas, policiais e bombeiros militares;

^†^Membros superiores do poder público, dirigentes de organizações de interesse público e de empresas, gerentes;

^‡^Profissionais das ciências e das artes;

^§^Técnicos de nível médio;

^//^Trabalhadores de serviços administrativos;

^¶^Trabalhadores dos serviços, vendedores do comércio em lojas e mercados;

^#^Trabalhadores agropecuários, florestais e da pesca;

**Trabalhadores da produção de bens e serviços industriais;

^††^Trabalhadores em serviços de reparação e manutenção.

Fonte: Elaboração própria.

O grupo ocupacional mais prevalente (29,2%) foi o de trabalhadores da produção de bens e serviços industriais, sendo a profissão de pedreiro a que mais foi registrada (23 notificações, podendo ir a 27 se incluirmos os serventes de obras). A segunda profissão mais prevalente entre os suicídios foi a de trabalhadores da agricultura, com 22 casos notificados (14 caseiros e 8 trabalhadores volantes). Em terceiro, verificamos a profissão de apontador de produção, com 20 notificações. O grupo sem ocupação definida (estudantes, dona de casa, aposentados/pensionistas e desempregados crônicos) apresentou 23,2% dos casos de suicídio com ocupação registrada.

O mapa na Figura 4 apresenta a distribuição espacial das taxas de suicídio em Chapecó (SC). Nota-se que na região extremo sul do município houve um agrupamento de 7 bairros com taxas altas, acima de 9,6 óbitos/100 mil hab. Ao norte, alguns bairros também apresentaram taxas nesse intervalo. O resultado sugere associação do suicídio com alta privação social.

**Figura 4:** Taxa de suicídio em Chapecó (SC), 2006 a 2019.

Fonte: Elaboração própria, com uso de dados do Instituto Brasileiro de Geografia e Estatística e Secretaria da Saúde de Chapecó.

## DISCUSSÃO

A taxa anual média padronizada de suicídio em Chapecó (SC) apresentou tendência de aumento no período analisado, sendo maior que a tendência nacional (p<0,001). A maioria dos suicídios foi devido a enforcamento, e as características com maior tendência de aumento de suicídio foram sexo masculino, faixa etária de 80 anos ou mais, escolaridade de 4 a 11 anos e estar solteiro. Em Chapecó (SC), a profissão de pedreiro/servente de obras foi a mais notificada entre os anos de 2006–2019, seguida dos trabalhadores da agricultura, e sua ocorrência concentra-se mais em regiões com alta privação social.

Historicamente, os estados do Sul apresentam taxas de suicídio mais altas que a média nacional, com maior concentração de óbitos na região^3,15,16^. Em Santa Catarina, a microrregião de Chapecó (SC) registra uma carga de suicídios superior à média estadual^17^.

A maioria dos óbitos por suicídio notificados entre 2006 e 2024 em Chapecó (SC) foi por enforcamento, estrangulamento e sufocação. Choi et al. trazem que o meio utilizado para efetuar o suicídio pode estar associado ao sexo e à idade^18^. Observou-se que homens com maior faixa etária escolhem formas mais violentas e letais, como enforcamento e disparo de arma de fogo, enquanto mulheres e jovens preferem formas mais sutis e lentas, como queda de altura e envenenamento.

Em um estudo conduzido no Rio Grande do Sul, o método mais utilizado para o suicídio foi o enforcamento^19^. Ressalta-se que o acesso a este método é de difícil controle, tornando-se um preditor de risco para violência autodirigida — por isso a importância da identificação precoce do público vulnerável para medidas preventivas, assim evitando que cheguem ao ato fatal.

Minayo et al.^20^, em estudo sobre uma análise do perfil epidemiológico dos homens idosos suicidas no Brasil, os dividiram em três ramos de ocupação: com maior percentual aparece a agricultura; e após, prestação de serviços, entre eles pedreiro e carpinteiro, corroborando o achado neste estudo. Traz, ainda, que os homens, criados e educados sob preceitos de masculinidade hegemônica, se sentem guardiões e que o suicídio é para eles uma saída honrosa de alguma dívida financeira, muito presente na ocupação como agricultor.

Em relação ao sexo, os resultados demonstraram uma maior tendência de suicídio na população masculina, representando cerca de 77% dos suicídios na cidade de Chapecó (SC). Dado esse que converge com estudo desenvolvido na cidade de Marabá (PA)^21^. Os achados confluem com o observado em nível nacional, podendo ser explicados devido à maior eficiência dos métodos escolhidos para consumar o ato.

Outro quesito importante sobre a população masculina seguir apresentando as maiores taxas de suicídio seriam os fatores socioeconômicos, incluindo instabilidades e comportamentos impulsivos e competitivos associados ao consumo de álcool — os quais podem se tornar preditores do ato suicida^22^.

Quanto à variável faixa etária, identificamos em Chapecó (SC) uma tendência de aumento a partir dos 20 anos de idade, e um aclive maior a partir dos 60 anos, com presença importante nos 80 anos ou mais. Foi observado no estado do Rio Grande do Sul, durante o período de 2010 a 2016, que ser do sexo masculino e ter idade superior a 45 anos eram preditores para o suicídio por meio do enforcamento; ainda, no período de 2009 a 2012, o sexo masculino permanecia como predisposto para este meio de execução do suicídio, associado a ser solteiro^16^. Os principais fatores de risco interligados com esses adultos mais velhos podem ser decorrentes de enfermidades físicas, afastamento social, problemas familiares, inatividade e uso abusivo de substâncias psicoativas — especialmente ligado a transtornos mentais, como a depressão. Esta última seria a que mais aflige esses adultos e idosos para mortes prematuras^16^.

A maioria dos indivíduos apresentava baixa/média escolaridade (4 a 11 anos), padrão também observado nacionalmente^22^. O grau de instrução, junto a outros fatores sociais, é um definidor do status econômico e social, influenciando diretamente nas preocupações e estresses diários, e por si em diante na autoestima e na ideação suicida^22^.

Sobre as tendências de suicídio de acordo com o estado civil, o município de Chapecó (SC) mostrou até o ano de 2009 que os casados possuíam maiores taxas de suicídio, porém, a partir de 2010, os solteiros aumentaram as referidas taxas, com tendência linear de aumento. No caso do Brasil, os solteiros sempre mantiveram tendência maior do que os casados e os viúvos durante todo o período analisado. Esse achado em Chapecó (SC) difere de pesquisa realizada no estado de Santa Catarina, nos anos de 2014 a 2018^23^, onde casados obtiveram porcentagem de 49,4% dos óbitos por suicídio, seguidos dos solteiros com 34,7%; já entre os demais, separados e divorciados somaram 10,1% e viúvos, 5,5%.

Chapecó (SC) apresenta tendência crescente de suicídios, maior que a nacional, possivelmente relacionada a fatores culturais, socioeconômicos e predominância de agroindústrias na economia local. Entre 2016 e 2022, as taxas de suicídio no Brasil também cresceram, período marcado por instabilidade política e reformas que afetaram a segurança socioeconômica dos trabalhadores. A flexibilização das relações de trabalho, as mudanças previdenciárias e as políticas de austeridade favoreceram um contexto de precarização laboral e a redução da proteção social. Esse fenômeno corrobora achados internacionais que associam crises econômicas e austeridade ao aumento do suicídio, sobretudo na ausência de mecanismos de proteção social, especialmente em países de média e baixa renda^24,25,26^.

No Brasil, o fenômeno pode relacionar-se não apenas ao desemprego, mas também às condições de trabalho e à perda de direitos, fatores que podem elevar o estresse psicológico mesmo entre trabalhadores formalmente ocupados^27^. Por sua vez, o decréscimo observado a partir de 2022 ocorre ainda em um contexto pandêmico tardio, tal como evidenciado em outros estudos^28,29^.

Estudo realizado na região da Amazônia Legal, marcada pela pecuária, monoculturas e atividade de extração, identificou maiores taxas de suicídio em áreas rurais^30^. O uso intensivo de agrotóxicos nesse modelo produtivo reforça a associação entre atividades agropecuárias, exposição ocupacional e maior ocorrência de suicídio^30,31^. Embora sejam necessários estudos para avaliar as relações causais, tais dados ecológicos em regiões com polos de produção agrícola, como é o caso do município de Chapecó (SC), podem sugerir uma possível associação entre trabalho e exposição a disruptores na agricultura.

Os agrotóxicos podem causar suicídio de forma direta ou indireta^31^. Diretamente, quando é ingerido ou por inalação proposital com a intenção de morrer; e de forma indireta, por sua utilização indevida, ocasionando efeitos no sistema nervoso no desenvolvimento de transtornos psíquicos, acarretando em desencadeamento de neuropatologias — e, por si, ideações suicidas^32^.

Ao avaliar a ocupação profissional dos casos de óbito por suicídio no período analisado, observou-se uma maior porcentagem quanto aos indivíduos envolvidos com a construção civil, em principal, pedreiros (29,2%); seguidos dos indivíduos sem uma ocupação definida (23,2%), como estudantes, donas de casa, desempregados, aposentados ou pensionistas; indivíduos com profissão definida como apontador de produção (13,1%); e trabalhadores da agricultura, com 11,1% dos achados.

Quanto aos indivíduos sem ocupação definida, é importante destacar que aposentados geralmente possuem experiência anterior ou continuam trabalhando para complementar a renda. Já a classificação de apontador de produção não pôde ser detalhada, pois não especifica objetivamente a função, embora se suponha ligação com trabalhadores de agroindústrias, especialmente frigoríficos de grande porte presentes no município.

Os achados convergem com a literatura ao indicar maior ocorrência de óbitos por suicídio entre trabalhadores da agricultura e da construção civil^33,34^. Essas ocupações são recorrentes entre as de maior vulnerabilidade ao comportamento suicida, sobretudo em contextos de precarização laboral, instabilidade econômica e elevadas exigências físicas e psicossociais.

O suicídio entre trabalhadores de setores marcados pela precarização laboral, como agricultura e construção civil, tem sido descrito em estudos sobre o perfil sócio-ocupacional dos óbitos. Nessas atividades, condições de trabalho vulneráveis podem intensificar o sofrimento mental e elevar o risco de suicídio, configurando um fenômeno observado em contextos nacionais e internacionais^35,36,37^.

Para Tornesi^38^, o assédio moral na construção civil é presente e, como fator de consequências à saúde, implica pensar nos danos psíquicos, que podem ser angústia, ansiedade, pensamentos tristes e recorrentes. O assédio pode trazer situações traumáticas, ou seja, o assediado não consegue superar o acontecido, podendo suscitar em uso de drogas, em especial o álcool. O sofrimento imposto e não compreendido pode ser causa de ideação suicida, porém esta não se limita a um fenômeno individual, mas sim a um processo social fruto de produções capitalistas^39^.

O estudo possui limitações inerentes ao delineamento ecológico, como a impossibilidade de inferência causal individual. A ausência de informações detalhadas sobre ocupação restringiu as análises à comparação das tendências de ocorrência; além disso, possíveis inconsistências e subnotificação podem ter reduzido a validade interna. Para minimizar esses efeitos, adotaram-se estratégias de padronização das variáveis e verificação da consistência dos registros. Apesar disso, os achados contribuem para a identificação de possíveis fatores associados ao suicídio e para nortear investigações futuras e estratégias preventivas voltadas às populações em risco.

Nesse sentido, as tendências observadas no município contribuem para traçar um perfil dos indivíduos com maior probabilidade de cometer suicídio: homens, idosos, trabalhadores da construção civil e/ou do setor agropecuário, solteiros e residentes em bairros com maior privação social.

Faz-se necessário frisar que, quanto à construção civil, em especial pedreiros, não há muitos estudos específicos nessa classe ocupacional, sendo que essa profissão apresenta alta frequência nos resultados também de outros estudos.

## MATERIAL SUPLEMENTAR

Figura Suplementar 1

Figura Suplementar 2

Figura Suplementar 3

## COMITÊ DE ÉTICA:

A presente pesquisa respeitou os princípios éticos, sendo aprovado pelo Comitê de Ética em Pesquisa da Universidade Federal da Fronteira Sul, sob o parecer n^o^ 3.907.939, aprovado em 10/03/2020.
