# Supplementary material for: ERRATUM: Suicide and occupation: spatial patterns in Chapecó (SC) and contextualization of the temporal trend in the national scenario, 2006–2024
Source: Rev Bras Epidemiol. 2026 Oct 5;29:e260030-ER. doi: 10.1590/1980-549720260030erratum (PMC13650568; doi:10.1590/1980-549720260030erratum)
Supplement: Translation

# Translation: ERRATA: Suicídio e ocupação: padrões espaciais em Chapecó (SC) e contextualização da tendência temporal no cenário nacional, 2006–2024

**DOI:** 10.1590/1980-549720260030.2erratum

No artigo *"Suicídio e ocupação: padrões espaciais em Chapecó (SC) e contextualização da tendência temporal no cenário nacional, 2006–2024*", DOI: https://doi.org/10.1590/1980-549720260030.2, publicado no periódico Rev Bras Epidemiol. 2026; 29: e260030.

**Onde se lia:**

**FONTE DE FINANCIAMENTO:** nenhuma.

**Leia-se:**

**FONTE DE FINANCIAMENTO:** Universidade Federal da Fronteira Sul - PES-2025-0260.
